# Physiological Tendon Thickness Adaptation in Adolescent Elite Athletes: A Longitudinal Study

**DOI:** 10.3389/fphys.2017.00795

**Published:** 2017-10-12

**Authors:** Michael Cassel, Konstantina Intziegianni, Lucie Risch, Steffen Müller, Tilman Engel, Frank Mayer

**Affiliations:** Department of Sports Medicine, University Outpatient Clinic, University of Potsdam, Brandenburg, Germany

**Keywords:** Achilles and patellar tendon, training adaptation, sonography, young athletes, non-athletes

## Abstract

Increased Achilles (AT) and Patellar tendon (PT) thickness in adolescent athletes compared to non-athletes could be shown. However, it is unclear, if changes are of pathological or physiological origin due to training. The aim of this study was to determine physiological AT and PT thickness adaptation in adolescent elite athletes compared to non-athletes, considering sex and sport. In a longitudinal study design with two measurement days (M1/M2) within an interval of 3.2 ± 0.8 years, 131 healthy adolescent elite athletes (m/f: 90/41) out of 13 different sports and 24 recreationally active controls (m/f: 6/18) were included. Both ATs and PTs were measured at standardized reference points. Athletes were divided into 4 sport categories [ball (B), combat (C), endurance (E) and explosive strength sports (S)]. Descriptive analysis (mean ± SD) and statistical testing for group differences was performed (α = 0.05). AT thickness did not differ significantly between measurement days, neither in athletes (5.6 ± 0.7 mm/5.6 ± 0.7 mm) nor in controls (4.8 ± 0.4 mm/4.9 ± 0.5 mm, *p* > 0.05). For PTs, athletes presented increased thickness at M2 (M1: 3.5 ± 0.5 mm, M2: 3.8 ± 0.5 mm, *p* < 0.001). In general, males had thicker ATs and PTs than females (*p* < 0.05). Considering sex and sports, only male athletes from B, C, and S showed significant higher PT-thickness at M2 compared to controls (*p* ≤ 0.01). Sport-specific adaptation regarding tendon thickness in adolescent elite athletes can be detected in PTs among male athletes participating in certain sports with high repetitive jumping and strength components. Sonographic microstructural analysis might provide an enhanced insight into tendon material properties enabling the differentiation of sex and influence of different sports.

## Introduction

Achilles and patellar tendinopathy was shown to be present already in adolescent athletes (Cook et al., 2000; Cassel et al., 2015). An increasing prevalence for patellar tendinopathy of 33% with increasing age up to 18 years could be identified (Simpson et al., 2016). In contrast, the Achilles tendon (AT) is less often affected during adolescence. Data among adolescent athletes from 16 different sports identified prevalence of Achilles and patellar tendinopathy with 1.8 and 5.8% at an average age of 13 years (Cassel et al., 2015). Athletes having patellar tendinopathy showed higher patellar tendon (PT) thickness compared to asymptomatic athletes in cross-sectional analysis (Cassel et al., 2015). Furthermore, a higher prevalence of intratendinous abnormalities (hypoechoic regions and higher grades of vascularization in ultrasound) was associated with patellar tendinopathy (Cassel et al., 2015). This supports the hypothesis of a continuum model of pathological tendon changes already at a young age (Cook and Purdam, 2009; Malliaras and Cook, 2011).

Besides a pathological increase in tendon diameter a physiological tendon adaptation due to repetitive and higher loading in sports is discussed (Kjaer et al., 2009; Malliaras and Cook, 2011; Cassel et al., 2016). ATs of healthy adult runners were shown to have higher cross-sectional area (CSA) compared to controls (Kongsgaard et al., 2005). Moreover, a correlation between body mass and AT-thickness was reported (Hirschmüller et al., 2010). For PTs, 12 weeks of strength training led to an increased CSA in Magnetic Resonance Imaging (MRI) scans of the proximal and distal tendon region by 4–7% in 12 untrained healthy males aged 25 years (Kongsgaard et al., 2007). Higher PT-CSA was also detected in the leading leg of seven elite fencers and badminton players with an average age of 23 years (Couppé et al., 2008). In a recent cross-sectional study on 500 adolescent athletes and 40 recreationally active controls with a mean age of 13 years, higher AT and PT thickness of ball and water sport athletes compared to controls could be detected (Cassel et al., 2016). However, statistically significant differences in AT and PT diameter for younger compared to older adolescent athletes could not be found (Cassel et al., 2016).

For the early detection of initial pathological changes normative values of physiological tendon thickness in athletes are required. Physiological AT diameter in adults has been reported to be 4–6 mm, while values of the patellar tendon thickness were observed between 3 and 5 mm, depending on measurement location (Schmidt et al., 2004; Fredberg et al., 2008; Hirschmüller et al., 2010; Cassel et al., 2012). Cross-sectional data among young adolescent athletes and non-athletes showed AT and PT thickness to be already on adult elite athletes' level (Cassel et al., 2016). Whether the higher tendon diameter in some types of sports has to be interpreted as a physiological adaptation due to loading or if it might represent the first sign of a pathological degeneration remains unclear (Malliaras and Cook, 2011; Cassel et al., 2016). None of the studies among asymptomatic adolescent athletes examined tendon dimensions as well as the presence of structural intratendinous abnormalities (i.e., echoic irregularities) potentially indicating initial tissue pathologies, in a longitudinal setting. In addition, the long-term effect of sex, training as well as anthropometric prerequisites on tendon thickness has to be determined.

The purpose of this study was to determine the longitudinal AT and PT thickness adaptation in healthy adolescent elite athletes compared to non-athletes during growth. In addition, the influence of sex, sport-specific loading, anthropometric data as well as intratendinous abnormalities were analyzed.

## Materials and methods

### Subjects

**Figure 1:**
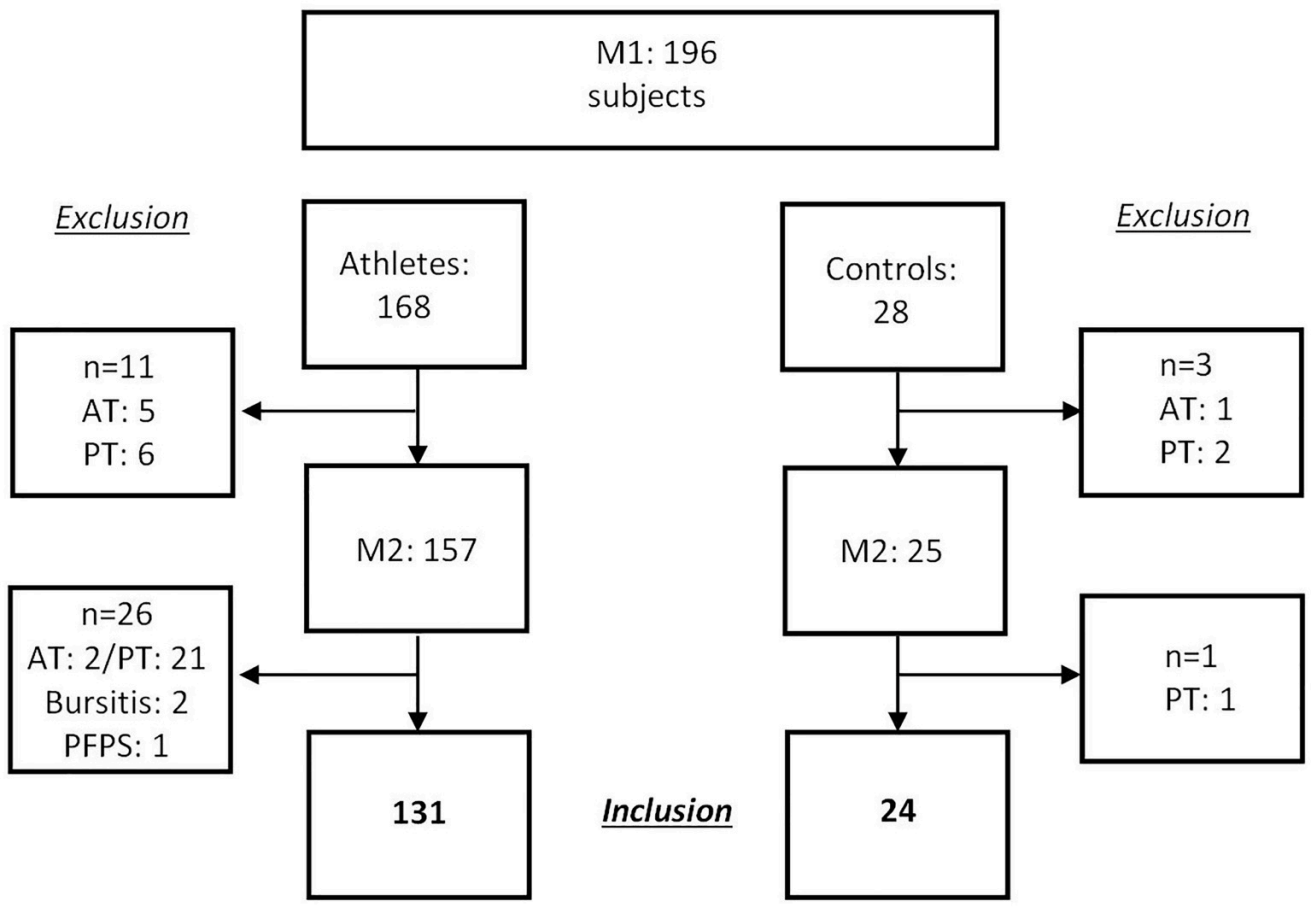
Study flow chart: recruitment at M1, exclusion of subjects due to diagnoses (Atp, Achilles tendinopathy; Ptp, patellar tendinopathy; PFPS, patellofemoral pain syndrome) in the region of Achilles and patellar tendons.

A total of 168 adolescent athletes were recruited in the context of a preparticipation examination before entrance to a federal elite school of sports (Mayer et al., 2012). In addition, 28 recreationally active control subjects were recruited from a local secondary school. Exclusion criteria were the presence of Achilles or patellar tendinopathy, rupture or surgery, rheumatic disease, dyslipidemia as well as acute injury of the knee or ankle. Due to the clinical presence of AT or PT tendinopathy at inclusion (first measurement day: M1) in 11 athletes and 3 controls, 14 subjects had to be excluded. In consequence, 157 healthy adolescent athletes out of 14 different sports and 25 healthy control subjects were included at M1 (Figure 1). Follow-up-examination (M2) took place after 3.2 ± 0.8 years for athletes and after 3.0 ± 0.1 years for controls. At M2 26 athletes and one control subject had to be excluded due to a newly developed diagnosis of tendinopathy in the AT or PT region (Figure 1). Parents of all adolescent athletes signed written informed consent before data acquisition. The study was approved by the Ethics Committee of the local University.

### Measurement procedure

Athlete's examination was conducted during preparticipation and annual health examinations at the university outpatient clinic, Olympic center, responsible for athletes' health in the federal state. For recreational subjects investigations took place at a secondary school by use of the same portable ultrasound equipment. Each examination composed of history, local tendon examination and a brief status of the adjacent joints. In addition to anthropometric data, age and sex, specific training-related data (sport-type, training duration and frequency) were documented in a standardized case report form.

Ultrasound scans were conducted to measure tendon diameter and to detect intratendinous abnormalities (intratendinous blood flow, hypo- and hyperechoic regions). Investigations of both ATs and PTs were performed using high-resolution ultrasonography (Viamo SSA-640A; Toshiba, Tokyo, Japan) with a multi-frequency linear transducer (PLT-704SY) and standardized transducer settings (11 MHz, gain = 93, DR = 50, penetration depth = 3 cm, focus at 0.5 cm). Ultrasound investigations were performed by three trained examiners. Examinations of ATs were investigated in a prone position with feet hanging over the examination table and ankle passively dorsi-flexed to a neutral position. PT examinations were carried out in a supine position with 30° knee flexion, controlled by use of a soft pad. As previously described, both ATs and PTs were longitudinally examined to determine tendon diameters at a reference point 2 cm proximal to the calcaneal insertion (AT 2 cm) and 2 cm distal the Patella (PT 2 cm), respectively (Cassel et al., 2012, 2016). Tendon thickness was reported to be reliably measureable at these standardized locations (Schmidt et al., 2004; Fredberg et al., 2008). Moreover, the diameter at the thickest part of the AT “mid-portion” was measured (AT max) (Cassel et al., 2012, 2016). Longitudinal scans were performed by a strict orthogonal placement of the transducer on ATs and PTs in order to measure “true tendon thickness”, excluding epitenon and paratenon.

For Doppler ultrasound (DU) detection of intratendinous blood flow (IBF) subjects were requested to relax their muscles leading to a slight plantar-flexion ankle angle, while the knee angle stayed at 30° flexion. IBF was measured using power Doppler ultrasonography (Advanced dynamic flow mode) with standardized DU settings (frequency = 4.5 MHz, pulse repetition frequency = 0.5 kHz, color velocity = 4.39 cm/s, color intensity just below the artifact threshold, size of the color box (region of interest, ROI = 3 cm^2^) (Cassel et al., 2015; Risch et al., 2016). IBF was graded according to a modified “Ohberg score” from 0 to 5 (0 = no vessels, 1 = 1–2 vessels, 2 = 3–5 vessels, 3 = vessels in up to 30%, 4 = vessels in 30–50% and 5 = vessels in >50% within the ROI) (Ohberg and Alfredson, 2002; Hirschmüller et al., 2010). Three examiners with experience in DU examinations performed the investigations.

### Data categorization and data analysis

Adolescent elite athletes were allocated into 4 categories according to their sports (ball sports (B, *n* = 40), combat sports (C, *n* = 39), endurance sports (E, *n* = 20) and explosive strength sports (S, *n* = 32); **Table 2**) (Müller et al., 2017). Data was analyzed descriptively by mean and standard deviation of anthropometrics, training and tendon parameters within all athletes (A), sports categories (B, C, E, S) and controls (Co). All tendon data is shown as means of both sides for each thickness parameter. Depending on data distribution (Kolmogorov-Smirnov-Test), differences were tested either by unpaired *t*-test, one-way and two-way ANOVA (*post-hoc* Tukey-Kramer-Test/Fishers least significant differences test) or Kruskal-Wallis-ANOVA (*post-hoc* Wilcoxon test) for non-normally distributed data. To identify potential influencing factors (age, sex, BMI, training amount and years, intratendinous abnormalities) on tendon thickness development a multiple logistic regression analysis was performed (Jmp 9.0 and SPSS Statistics 20). Results with a *p* < 0.05 were considered significant.

## Results

A total of 131 athletes as well as 24 controls were analyzed (Table 1). Anthropometric and training data of athletes, controls and sports categories are presented in Table 2. Athletes showed higher height, weight, BMI and training amount at M2 compared to M1 (*p* < 0.001). Controls presented higher anthropometric data at M2 (*p* < 0.003), but did not show statistically significant differences for training data (*p* > 0.05). At both measurement days, athletes were younger and had higher training volume than controls (*p* < 0.001; Table 2).

**Table 1:** Number of athletes [n] as well as sex distribution [n] per sport categories (ball sports [B], combat (C), endurance [E] and explosive strength sports [S]), controls (Co), and group/sport at M2.

**Category**	**n**	**n**	**Group/Sport**	**n**	**n**
		**(m/f)**			**(m/f)**
Co	24	6/18	Controls	24	6/18
B	40	33/7	Handball	10	9/1
			Soccer	27	24/3
			Volleyball	3	0/3
C	39	30/9	Boxing	5	4/1
			Judo	19	13/6
			Wrestling	15	13/2
E	20	13/7	Canoeing	8	6/2
			Cycling	2	2/0
			Rowing	3	2/1
			Swimming	7	3/4
S	32	14/18	Modern Pentathlon	5	3/2
			Shooting	2	2/0
			Track and field	25	9/16

**Table 2:** Anthropometric and training data among all athletes (A), controls (Co) and sport categories (ball sports [B], combat (C), endurance [E], and explosive strength sports [S]) at M1/2.

**Category**	**M**	**N (m/f)**	**Age [years]**	**Height [cm]**	**Weight [kg]**	**BMI [kg/m^2^]**	**Training [h/week]**	**Training years**	**M1/M2 [*p*-value]**
A	M1	131 (90/41)	12.1 ± 0.7	157 ± 9	45 ± 9	18.1 ± 2.5	5.8 ± 3.0^*^	4.3 ± 2.3^*^	<0.001
	M2	131 (90/41)	15.2 ± 0.7	174 ± 9^*^	63 ± 11	20.8 ± 2.4	14.4 ± 4.4^*^	7.5 ± 2.5^*^	
Co	M1	24 (6/18)	13.3 ± 0.6^*^	160 ± 8^*^	48 ± 12	18.8 ± 3.4	1.9 ± 1.3	2.5 ± 2.8	^+^≤ 0.003
	M2	24 (6/18)	16.2 ± 0.6^*^^+^	169 ± 9^+^	60 ± 14^+^	21.0 ± 3.3^+^	2.4 ± 2.2	3.9 ± 3.3	
B	M1	40 (33/7)	12.0 ± 0.5	157 ± 8	45 ± 7	18.0 ± 1.6	5.2 ± 1.8	5.7 ± 2.0	<0.001
	M2	40 (33/7)	15.1 ± 0.6	175 ± 8	64 ± 10	20.7 ± 2.2	12.2 ± 3.2	8.8 ± 2.0	
C	M1	39 (30/9)	12.1 ± 0.9	154 ± 10	47 ± 12	19.3 ± 3.2	6.1 ± 2.7	4.2 ± 2.3	<0.001
	M2	39 (30/9)	15.2 ± 0.8	170 ± 9	62 ± 12	21.5 ± 2.8	15.2 ± 4.3	7.1 ± 2.6	
E	M1	21 (14/7)	12.0 ± 0.8	160 ± 8	46 ± 8	17.6 ± 1.8	7.7 ± 3.9	4.1 ± 2.2	<0.001
	M2	20 (13/7)	15.2 ± 1.0	180 ± 10	68 ± 11	20.9 ± 2.2	17.1 ± 3.2	7.5 ± 2.3	
S	M1	31 (13/18)	12.2 ± 0.6	158 ± 8	43 ± 9	17.0 ± 2.3	4.9 ± 3.3	3.0 ± 2.0	<0.001
	M2	32 (14/18)	15.3 ± 0.5	174 ± 7	61 ± 9	20.2 ± 2.3	14.5 ± 5.3	6.3 ± 2.3	
*p*-value^#^	M1	131 (90/41)	n. s.	n. s.	n. s.	≤0.001	≤0.002	<0.001	
	M2	131 (90/41)	n. s.	<0.001	n. s.	n. s.	<0.001	<0.001	

* Represents statistically significant higher value in comparison of A against Co at M1/2 (p < 0.05 for height; p < 0.001 for age, training h/week and years)

+ *Represents statistically significant higher values of Co-subjects at M2*.

# *Data of p-value for ANOVA of differences between sports categories at M1 and M2 (n. s.: not significant). Post-hoc tests: Height: p ≤ 0.04: B/E/S higher C and E higher S at M2; BMI: p ≤ 0.006 for B/C higher S at M1; training h/week: p < 0.008 for E higher B/S and C higher S at M1, p ≤ 0.03 for C/E/S higher B and E higher S at M2; training years: p ≤ 0.03 for B higher C/E/S and C higher S at M1, p < 0.03 for B higher C/E/S at M2*.

### Tendon parameters at M1 and M2 (cross-sectional analysis)

Athletes had higher AT thickness than controls at M1 and M2 (*p* < 0.001) as well as PT thickness at M2 (*p* ≤ 0.04; Table 3). Sports categories showed highest AT thickness of B (AT 2 cm at M1, AT max at M1/2) and highest PT thickness of E at M1 (*p* < 0.04), while there were no statistically significant differences of PT-thickness at M2 (*p* > 0.05). Male subjects had higher AT and PT thickness compared to females at both measurement days among athletes and controls (*p* < 0.05; Table 4). Intratendinous abnormalities were present at both measurement days. In M1 IBF (grade 1 and 2) was visible in 5% of PTs, hypoechogenicities in 1.5% of ATs and 2% of PTs. In M2 IBF (grade 1 and 2) was visible in 3% of ATs and 4% of PTs, hypoechogenicities in 2.5% of ATs and 8% of PTs, hyperechogenicities in 1% of ATs as well as PTs.

**Table 3:** Mean Achilles and patella tendon thickness values [mm] of all athletes [A], controls [Co] and different sport categories (ball sports [B], combat (C), endurance [E], and explosive strength sports [S]) at M1/2 (Tendon values for Achilles tendon 2 cm proximal to the insertion [AT 2 cm] and maximum midportion value [AT max] and for Patella tendon 2 cm distal to the tendon insertion [PT 2 cm]).

**Location**	**M**	**A**	**Co**	**B**	**C**	**E**	**S**	**B/C/E/S**
	***n***	**[*n* = 131]**	**[24]**	**[40]**	**[39]**	**[21/20]**	**[31/32]**	***p*-value^#^**
AT 2 cm	M1	5.1 ± 0.6^+^	4.6 ± 0.6	5.2 ± 0.6	5.0 ± 0.7	5.3 ± 0.7	5.1 ± 0.5	n. s.
	M2	5.1 ± 0.6^+^	4.6 ± 0.4	5.3 ± 0.5^#^	5.0 ± 0.7	5.0 ± 0.6	5.0 ± 0.6	<0.04
AT max	M1	5.6 ± 0.7^+^	4.8 ± 0.4	5.8 ± 0.6^#^	5.4 ± 0.6	5.7 ± 0.9	5.5 ± 0.5	=0.057
	M2	5.6 ± 0.7^+^	4.9 ± 0.5	5.9 ± 0.6^#^	5.6 ± 0.9	5.5 ± 0.6	5.4 ± 0.6	≤0.03
PT 2 cm	M1	3.5 ± 0.5	3.4 ± 0.5	3.5 ± 0.4	3.6 ± 0.5	3.8 ± 0.5^#^	3.3 ± 0.4	≤0.01
	M2	3.8 ± 0.5*^+^	3.5 ± 0.5	3.9 ± 0.5^*^	3.9 ± 0.6^*^	3.7 ± 0.4	3.6 ± 0.4^*^	n. s.
M1/M2 [*p*-value]^*^		<0.001	n. s.	<0.001	<0.02	n. s.	<0.02	

* *Data of t-test (statistical significant differences M1 vs. M2) for values of each parameter (location) in different categories (n. s.: not significant)*.

+ *Represents statistically significant differences of A vs. Co at both measurement days for AT 2 cm and AT max (p < 0.001) and for PT 2 cm at M2 (p ≤ 0.04)*.

# *ANOVA p-value for sports categories at M1 as well as at M2. Post-hoc tests: AT 2 cm: p < 0.04 for B higher C/E/S at M2; AT max: p ≤ 0.01 for B higher C at M1; p < 0.03 for B higher S at M2; PS 2 cm: p < 0.01 for E higher S at M1*.

**Table 4:** Mean Achilles (AT 2 cm, AT max) and patella tendon thickness (PT 2 cm) values [mm] for sex [m/f] among all athletes [A] and controls [Co] at M1/2.

**Category**	**M**	**AT 2 cm**		**AT max**		**PT 2 cm**		**M1-M2 [*p*-value^#^]**
	**Sex**	***m***	***f***	***m***	***f***	***m***	***f***	
A	M1	5.2 ± 0.5^+^	4.9 ± 0.7^*^	5.7 ± 0.7^+^	5.3 ± 0.7^*^	3.6 ± 0.5^+^	3.3 ± 0.4	<0.001
	M2	5.2 ± 0.6^+^	4.7 ± 0.6	5.8 ± 0.7^+^	5.2 ± 0.6^*^	4.0 ± 0.5^+^^#^	3.4 ± 0.3	
Co	M1	5.2 ± 0.6^+^	4.4 ± 0.4	5.4 ± 0.7^+^	4.6 ± 0.3	3.8 ± 0.6^+^	3.2 ± 0.4	n. s.
	M2	4.9 ± 0.7^+^	4.5 ± 0.4	5.4 ± 0.5^+^	4.7 ± 0.4	4.0 ± 0.5^+^	3.4 ± 0.4	
*p*-value^*^	M1	n. s.	≤0.002	n. s.	<0.001	n. s.	n. s.	
	M2	n. s.	n. s.	n. s.	≤0.01	n. s.	n. s.	

* *Represents statistically significant higher value in comparison of A against Co at M1 and M2*.

+ *Represents statistically significant sex differences within groups (A or Co) at M1 and M2 for AT 2 cm (A: p ≤ 0.02; Co: p < 0.05) and AT max (A: p < 0.001; Co: < 0.01) and for PT 2 cm (A: p < 0.001; Co: < 0.01)*.

# *Represents statistically significant higher value of PT 2 cm in male athletes at M2 compared to M1 (p < 0.001). All other tendon parameters of A or Co between M1 and M2 were not statistically significant (p > 0.05)*.

### Tendon adaptation (longitudinal analysis)

**Figure 2:**
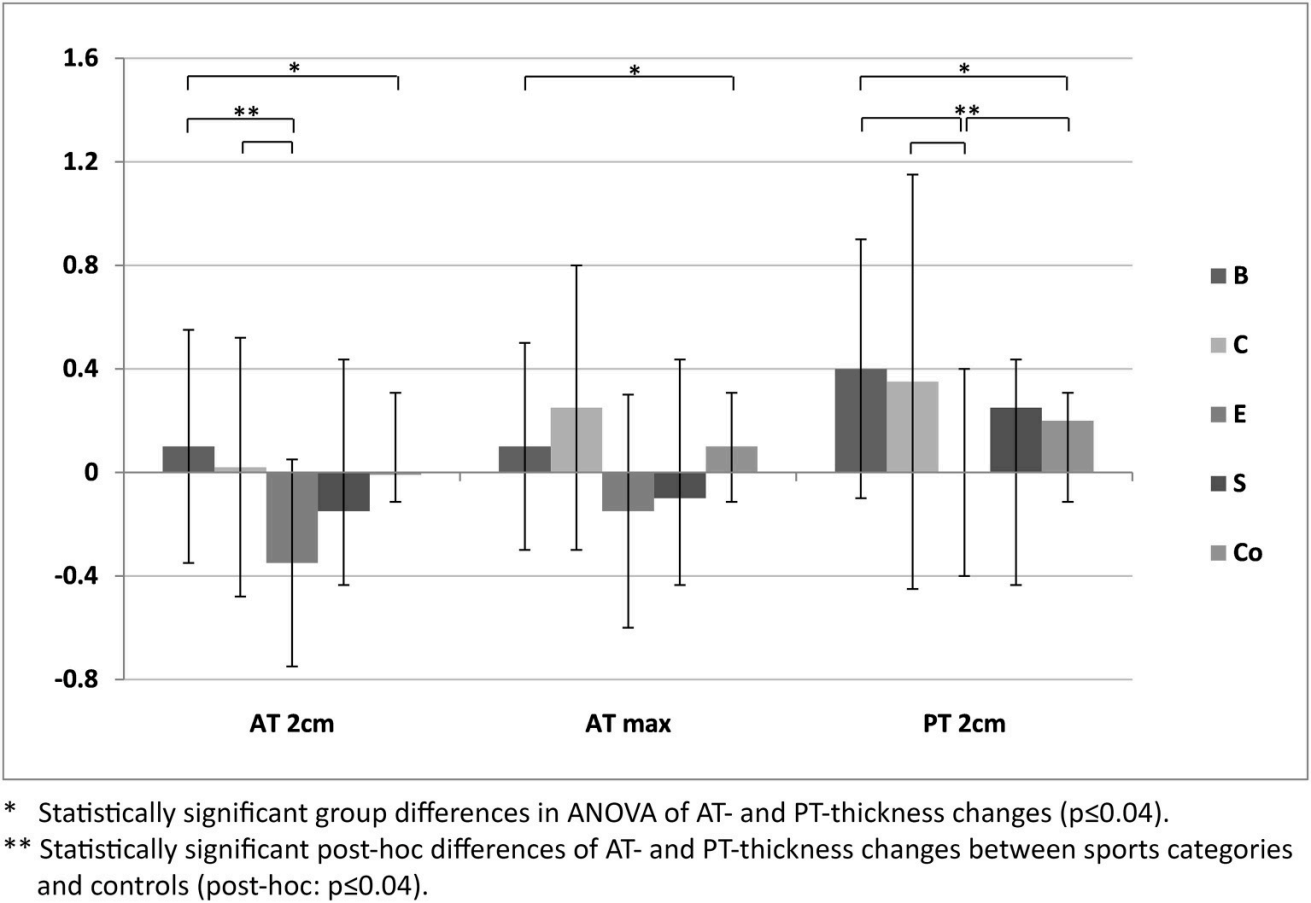
Differences of mean Achilles (AT 2 cm, AT max) and patellar tendon thickness (mean ± SD [mm]) of M2-1 between sport categories (ball sports [B], combat [C], endurance [E] and explosive strength sports [S]), and controls (Co).

**Figure 3:**
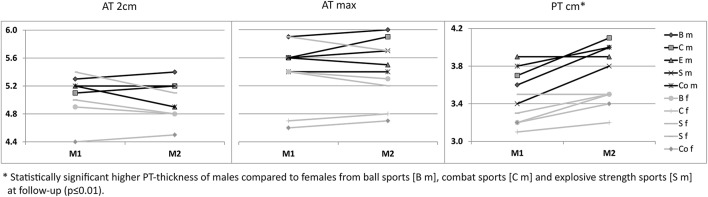
Sex-specific (m/f) mean Achilles (AT 2 cm, AT max) and patellar tendon thickness [mm] from M1 to M2 between sport categories (ball sports [B], combat [C], endurance [E] and explosive strength sports [S]), and controls (Co).

For ATs, thickness did not differ statistically significant between M1 and M2, neither for athletes or controls, nor between different sport categories (*p* > 0.05; Table 3). For PTs, athletes presented higher thickness at M2 compared to M1 (*p* < 0.001). In contrast, controls did not show statistically significant differences between measurement days (*p* > 0.05; Table 3). Differences (M2-1) of AT- and PT-thicknesses between sports categories and controls ranged from –0.3 mm to 0.4 mm (Figure 2). Male athletes had higher PT thickness at M2 (*p* < 0.001). Sex-specific analysis of sport categories identified male subjects from B, C and S having higher PT-thickness at M2 (*p* ≤ 0.01; Figure 3). Regression analysis showed an influence of sex on all tendon thickness parameters at both measurement days in athletes and controls, with males having higher thicknesses than females (*p* < 0.05). Increased PT thickness was associated with higher BMI in athletes. In contrast, factors age, training amount (h/week and years) and presence of intratendinous abnormalities did not lead to a higher AT or PT thickness (*p* > 0.05).

## Discussion

The study aimed to determine the physiological AT and PT thickness adaptation in healthy adolescent elite athletes in comparison to non-athletes during growth. Furthermore, the influences of sex, sport-specific loading, anthropometric data as well as intratendinous abnormalities were analyzed.

AT-thickness did not differ significantly between measurement days, neither for athletes nor for controls. In addition, AT-thickness was shown to be on adult athletes' level (Hirschmüller et al., 2010; Cassel et al., 2016), indicating that maximum AT thickness is already determined by the age of 12 years. It was previously argued that an increased tendon thickness results from an adaptation to increased force generation capacity, i.e., due to higher body/muscle mass, leading also to increased stiffness of its structure (Intziegianni et al., 2016; Kulig et al., 2016). Taking results of the present study into account it can be hypothesized that tendons might adapt to higher forces by changes on micro-morphological level (Kulig et al., 2016), leading to higher fiber density, which was not analyzed in the present study. Further investigations by use of tendon tissue characterization methods (i.e., spatial frequency parameters) might clarify this possible relationship (Bashford et al., 2008; van Schie et al., 2010).

In contrast, an increase in PT-thickness could be detected with up to 10% differences in athletes but not in controls. This supports the hypothesis of a physiological tendon adaptation by thickness due to loading. However, thickness adaptation seems to be dependent on sports categories as well as sex, as an increase was shown to be solely present in males from ball, combat and explosive strength sports. In the same line, a cross-sectional study including 500 adolescent athletes and 40 non-athletes previously did not show a general physiological AT and PT adaptation related to tendon loading (Cassel et al., 2016). Comparable to present longitudinal data, cross-sectional results identified small differences (up to 10% change) in PT thickness among athletes of certain sport categories (i.e., ball sports) compared to controls (Cassel et al., 2016). Further data analyzing the physiological thickness adaptation in young adolescent athletes is recently not available. During late adolescence (16–18 years of age), an increase up to 27% in PT-CSA has been reported in the leading leg among 18 Volleyball players (Mersmann et al., 2017). However, presence or absence of structural intratendinous abnormalities have not been considered in this study (Mersmann et al., 2017). In adult athletes, sports-specific PT adaptation by thickness has also been postulated (Kongsgaard et al., 2007; Couppé et al., 2008; Seynnes et al., 2009). Couppé et al. found higher PT-CSA in leading leg of 7 pain-free fencing and badminton elite athletes compared to the contralateral side using MRI (Couppé et al., 2008). Seynnes et al. examined PT-CSA in 15 young men (mean age 20 years) before and after 9 weeks of resistance training and reported an increase in CSA of approximately 4% (Seynnes et al., 2009). Kongsgaard et al. investigated PT-CSA in 12 untrained male participants before and after a 12 weeks strength training program. Participants presented a 4–7% higher tendon CSA at the distal and proximal tendon insertion (Kongsgaard et al., 2007). In contrast, Kubo and Yata did not see a change in patellar tendon CSA in 9 healthy males (mean age 21 years) following 3 month of resistance training (Kubo and Yata, 2017). Changes in CSA as well as sample sizes of these investigations are very small and should therefore be interpreted with caution. However, data are in line with results of the present study since detected changes are small and only visible in PTs among athletes of specific sports categories.

### Influence of pathology

Existence of intratendinous abnormalities (i.e., hypoechogenicities) usually goes along with enlarged tendon diameter (Docking et al., 2015) and has been reported to predict patellar tendinopathy in athletes (Gisslén et al., 2007; Comin et al., 2013; Visnes et al., 2015). Malliaras and Cook stated that mild patellar tendon thickening (>4.2 mm among men, >4.0 mm among women) may already indicate pathology among active athletes (Malliaras and Cook, 2011). The present study is the first differentiating between tendons with and without structural irregularities when looking at tendon thickness development. However, regression analysis did not show a statistically significant influence of intratendinous abnormalities on tendon thickness, neither in athletes nor in controls. Furthermore, within subgroups of sport categories mean PT thickness values were reported to be below the thickening threshold, postulated by Malliaras and Cook (2011).

In a recently published longitudinal study Visnes et al. followed 141 initially asymptomatic young elite volleyball players (in mean 17 years of age at inclusion) for an average of 1.7 years (Visnes et al., 2015). A total of 22 players developed tendinopathy. The remaining 119 athletes did not show a statistically significant change in PT diameter (differences 0.0–0.1 mm) at follow-up. However, mid-tendon PT-thickness was already on a high level (males: 4.3 mm, females: 3.7 mm). Furthermore, 50% of asymptomatic volleyball players already had intratendinous abnormalities at baseline (Visnes et al., 2015). These findings support data of the present study showing that PT thickness is already determined in earlier stages of adolescence. Moreover, changes in thickness or physiological adaptation by thickness are small and structural changes have to be considered when interpreting physiological adaptations.

### Influence of sex

Present longitudinal data show higher PT-thickness in follow-up exclusively in males from ball, combat and explosive strength sports suggesting a sex-specific adaptation of PTs due to loading. Sex-influence on absolute thickness values was also detected for AT tendon thickness parameters at both measurement days, irrespective of activity level in athletes as well as in controls. This is in line with data from Visnes et al. who found PT and quadriceps tendon thickness to be statistically significantly higher in 16–20 year old male compared to female volleyball players (Visnes et al., 2015). This fact might be explained by hormonal levels or genetic prerequisites. Increased estrogen levels were seen to be responsible for inhibition of the acute exercise-related collagen synthesis (Magnusson et al., 2007; Kjaer et al., 2009; Hansen and Kjaer, 2016). However, in the present study, lower AT and PT thickness of females were already detected at baseline (mean age of 12 years), where hormonal influences are assumed to be less relevant. Furthermore, differences were visible irrespective of activity level in athletes as well as controls.

### Limitations

When interpreting the results, some limitations have to be considered. Sample size of the longitudinal study was relatively low, especially for the differentiation of several sports disciplines. Furthermore, measurement location of thickness parameters is assumed to be of high relevance when interpreting physiological tendon adaptation by thickness, especially during maturation. According to prior sonographic reliability studies thickness parameters were used at presented sites instead of width or CSA-measurements (Fredberg et al., 2008; Cassel et al., 2012). For ATs, a second measurement location (thickest part in midportion) was chosen to consider possible length changes due to maturation. For PTs, it was decided to take only one standardized parameter in the “mid-tendon” since tendinopathic tissue abnormalities in adolescent athletes usually are visible in the proximal or distal portion of the tendon (Cassel et al., 2015). However, since some studies reported on an increased thickness or CSA in proximal and/or distal PT region following training (Kongsgaard et al., 2007; Couppé et al., 2008; Kulig et al., 2013) future investigations should also consider the insertional regions. Additionally, in order to take length changes due to maturation into account, future investigations might also include measurement of thickest mid-tendon part. Overall, three trained examiners were responsible for the ultrasound measurements, which might have influenced the results. However, investigations on intra- and inter-observer reliability of AT and PT thickness measurements as well as IBF assessment using the ‘modified Ohberg Score’ showed good to high reliability (Fredberg et al., 2008; Risch et al., 2016).

## Conclusion

In between 12 and 15 years of age AT-thickness did not increase due to loading. In contrast, PT-thickness was increased in male athletes among ball, combat and explosive strength sports, indicating a sports- and sex-specific tendon adaptation by thickness. However, changes seem to be small. Controversial adaptability of ATs and PTs could explain the higher sustainability of young athletes in the development of patellar tendinopathy (Mersmann et al., 2016). Influence of sex is obvious in all parameters measured at both measurement days. Implementation of sonographic microstructural analysis might provide an enhanced insight into tendon material properties enabling the differentiation of sex and influence of different sports.

## Author contributions

The author contributions are distributed as followed: conception or design of the work (MC, SM, FM), data acquisition (MC, LR), data analysis (MC, KI) and interpretation (MC, KI, LR, TE, FM); drafting (MC) or revising (MC, KI, LR, SM, TE, FM) the work; final approval of the version to be published (MC, KI, LR, SM, TE, FM); agreement to be accountable for all aspects of the work (MC, KI, LR, SM, TE, FM).

### Conflict of interest statement

The authors declare that the research was conducted in the absence of any commercial or financial relationships that could be construed as a potential conflict of interest.
